# Biochemical profile differences during the transition period based on different levels of non-esterified fatty acids at 7 weeks before parturition in Mediterranean Italian dairy buffaloes (*Bubalus bubalis*)

**DOI:** 10.3389/fvets.2024.1404041

**Published:** 2024-07-02

**Authors:** Anastasia Lisuzzo, Elisa Mazzotta, Giovanna Cappelli, Alessandra Martucciello, Bruno Moura Monteiro, José Nélio Sousa Sales, Gabriele Di Vuolo, Immacolata De Donato, Lorena Schiavo, Esterina De Carlo, Pasquale Rossi, Barbara Contiero, Enrico Fiore, Domenico Vecchio

**Affiliations:** ^1^Department of Animal Medicine, Production, and Health, University of Padova, Legnaro, PD, Italy; ^2^Istituto Zooprofilattico Sperimentale delle Venezie, Legnaro, PD, Italy; ^3^Istituto Zooprofilattico Sperimentale del Mezzogiorno, National Reference Center on Water Buffalo Farming and Productions Hygiene and Technologies (CReNBuf), Portici, Italy; ^4^Institute of Animal Health and Production (ISPA), Federal Rural University of the Amazon (UFRA), Belém, PD, Brazil; ^5^Department of Veterinary Medicine, Federal University of Lavras, Lavras, MG, Brazil; ^6^Sud Rienergy Agricultural Society S.r.l., Corigliano Calabro, Italy

**Keywords:** metabolic changes, oxidative stress, lipomobilization, buffaloes, transition period

## Abstract

Metabolic adaptations to negative energy balance, as well as lipomobilization, influence inflammatory responses, immune function, and oxidative stress in animals. This study aimed to evaluate the biochemical profile of Mediterranean buffaloes with different levels of lipomobilization from the prepartum to the postpartum period. A total of 76 Mediterranean buffaloes were enrolled, and a weekly blood sample was taken from 7 weeks before to 6 weeks after calving. The concentration of non-esterified fatty acids (NEFAs) was determined in serum and was used to categorize buffaloes into three lipomobilization groups 7 weeks before calving: mild (NEFA-I; NEFA ≤ 0.29 mEq/L; *n* = 18), medium (NEFA-II; 0.29 < NEFA < 0.57 mEq/L; *n* = 20), and severe (NEFA-III; NEFA ≥ 0.57 mEq/L; *n* = 38). Two-way repeated measures ANOVA was used to assess changes within and between the groups and over time. Significant differences were found in the concentration levels of NEFA, β-hydroxybutyrate, glucose, cholesterol, protein profile, oxygen radicals, antioxidants, lysozyme, complement, and minerals. These results suggest that both medium and severe lipomobilization groups are associated with metabolic alterations. In conclusion, buffaloes with higher NEFA levels (>0.29 mEq/L; NEFA-II and NEFA-III) at 7 weeks before calving should be monitored more closely to reduce the risk of metabolic diseases. Furthermore, the medium (NEFA-II) and severe (NEFA-III) lipomobilization groups could be associated with differences in the animals’ ability to manage their metabolic status. Specifically, the severe mobilization group was most associated with a greater energy deficit during both the prepartum and postpartum periods without oxidative stress. On the contrary, the medium mobilization group was associated with a less severe energy deficit but was also associated with an inflammatory status and oxidative stress during the prepartum period. These distinctions highlight the need for tailored management strategies to address varying levels of metabolic stress in dairy buffaloes.

## 1 Introduction

The world population of buffaloes (*Bubalus bubalis*) is estimated at 205 million, reared across more than 66 countries; this represents a 24.89% increase from the year 2000 to 2022 (1).

World buffalo milk production is estimated to have reached 143 million tons in 2022, marking a 49% increase from 2011 (1). The largest herds of buffalo are found in developing countries such as India (54.53%), Pakistan (21.29%), China (13.10%), and Nepal (2.50%) (1). These herds are predominantly managed through family-owned and extensive farming systems. Buffalo’s fresh milk represents more than 15% of the total global production of whole fresh milk in 2022 (Table 1), and this percentage increased to 34% within Asia (Table 1). In Europe, 89% of buffalo are bred in Italy, where the buffalo population has grown from 12,000 heads in 1947 to 435,972 in 2023 (3). Italy boasts 2,381 farms, with over half (50.48%) located in the Campania region. This concentration has positioned Italy as the seventh largest producer of fresh buffalo milk in the world, significantly benefitting economically from the production of Mozzarella di Bufala Campana Dop cheese, which generated 893 million euros in 2023 (4). Another important aspect is related to the intensification of the Italian breeding system, with 39% of the animals raised on farms with more than 500 heads (3). The greater interest in buffalo products, such as milk and meat, has increased the transition from farmyard activity to commercial holdings and large herds, which is more similar to the dairy cattle industry (5). However, the use of techniques that have not always been developed specifically for buffalo species can increase the incidence of various conditional diseases (6). Late gestation and early lactation, which is also known as the transition period, represent the time of the highest incidence of metabolic and reproductive diseases (7).

**Table 1:** Global production of whole fresh milk by dairy species (buffalo, cow, goat, sheep, and camel) for each continent in 2022.

Whole fresh milk in 2022^*^		Buffalo	Camel	Cow	Goat	Sheep	Total
World	tons	143.573.178	4.116.669	753.320.577	19.191.572	10.093.015	930.295.011
	% on area	15.43%	0.44%	80.98%	2.06%	1.08%	100.00%
Asia	tons	142.012.348	1.239.814	258.554.636	10.841.780	4.513.333	417.161.911
	% on area	34.04%	0.30%	61.98%	2.60%	1.08%	100.00%
America	tons	NA	NA	199.278.256	828.039	92.085	200.198.380
	% on area	NA	NA	99.54%	0.41%	0.05%	100.00%
Europe	tons	428.830^**^	28	226.068.632	3.057.009	3.081.490	232.635.989
	% on area	0.18%	0.00%	97.18%	1.31%	1.32%	100.00%
Oceania	tons	NA	NA	30.706.258	39.89	NA	30.706.298
	% on area	NA	NA	100.00%	0.00%	NA	100.00%
Africa	tons	1.276.000	2.876.825	39.896.283	4.464.704	2.406.106	50.919.918
	% on area	2.51%	5.65%	78.35%	8.77%	4.73%	100.00%

^*^Data from 2022 (1). ^**^Data integrated by FAO (1) and CRenBuf (2).

The percentage (%) of animal species milk was calculated according to each geographical area.

NA, Not available.

In particular, the imbalance between energy intake and energy demand resulted in a para-physiological negative energy balance (NEB) in dairy ruminants (8, 9). Body resources, such as muscle, adipose, and bone tissues, are then mobilized in the form of amino acids, non-esterified fatty acids (NEFAs), and calcium to support energy requirements for the growth of the fetus and the onset of lactation (7). These metabolic adaptations are also accompanied by alterations in inflammatory responses that modify immune function and may generate oxidative stress in animals (10). Therefore, the mobilization of body resources can increase the risk of several interrelated diseases, although a certain degree of immune activation and inflammation is usually found in periparturient animals. These diseases have a negative impact on the growth, health, lactation, and reproductive performance of the animals (7, 11, 12).

The body condition score (BCS) has been proposed to estimate the energy balance and body reserves instead of variations in live weight (6). However, NEFA appears to be more sensitive to energy balance (11). A NEFA concentration above 0.29 mEq/L in the prepartum period or 0.57 mEq/L in the postpartum period has been identified as a critical threshold for an increased risk of metabolic diseases in dairy cows (13). Along with NEFA, β-hydroxybutyrate (BHB), one of the main ketone bodies produced by fatty acid oxidation, is also a common indicator of NEB during the transition period, with a threshold of 1.0 mmol/L in the postpartum period of cows (9, 14). However, a specific threshold for NEFA and BHB in buffaloes has not yet been established, and the dairy cows’ cut-offs of these parameters are frequently applied in this species (15).

The hypothesis is that greater lipomobilization could cause more severe alterations in the biochemical profile of buffaloes. To date, there is very limited information relating to metabolic profile curves in the buffalo species during the transition period in intensive farming, and in particular, given its tropical origin, it could present qualitative and quantitative variations that do not overlap with what was found in dairy cattle. Therefore, this study aimed to evaluate the biochemical profile of Mediterranean buffaloes with different levels of lipomobilization from the prepartum period to the postpartum period.

## 2 Materials and methods

### 2.1 Ethics statement

The animal studies were approved by the Italian Ministry of Health (authorization number. 758/2017-PR). The studies were conducted in accordance with the local legislation and institutional requirements. Written informed consent was obtained from the owners for the participation of their animals in this study.

### 2.2 Animals

A total of 76 primiparous and multiparous Mediterranean buffaloes of a mean (mean ± standard error) age of 2,236 ± 106 days, a parity order between 1° and 7°, a mean body condition score of 7.4 ± 0.07, and a previous lactation with a mean duration of 289 ± 7 days and 2,994 ± 70 kg mean milk production were enrolled from a commercial farm in the Cosenza province (Calabria, Italy) for 11 months, from July to June. All animals underwent fixed time assisted insemination. The buffaloes were housed in open yards, providing each animal with 15 m^2^ of space and a covered bedding area of 7 m^2^ per head. The feeder space allocated per head was 0.75 m. A total mixed ratio (TMR) was provided twice daily, adjusted for a 10% feed refusal rate (as-fed basis), and water was available *ad libitum*. The chemical composition of the TMR changed according to the reproductive phase of the animals: late lactation, dry-off period, and early lactation (Table 2). The animals were dried off 90 days before the estimated calving date and underwent the clinical examinations conducted by veterinarians during the study. In particular, at the time of drying off, each buffalo underwent a clinical examination that included confirmation of gestation, an assessment for the absence of external lesions, an evaluation of nutritional status, and an examination of udder quarters using the standard California mastitis test. During weekly sampling, any problems during delivery and postpartum were recorded: dystocia, abortions, placenta retention, stillbirth, uterine and vaginal prolapses, endometritis and metritis, and clinical mastitis. Only buffaloes that were clinically healthy at 7 weeks before the estimated calving date were enrolled in this study. During the trial, the BCS was assessed using the method described by Wagner et al. (16), which was adapted to a 10-point scale for detailed evaluation (17).

**Table 2:** Chemical composition of the total mixed ratio (TMR) according to the reproductive phase.

Units	Late lactation	Dry-off period	Early lactation
	DM%^1^	DM%^1^	DM%^1^
UFL^2^	0.88	0.64	0.92
CP^3^	14.67	8.92	15.43
PD^4^	6.82	0.86	7.36
PDIN^5^	10.14	5.98	10.68
PDIE^6^	9.78	6.87	9.98
PDIA^7^	5.25	2.52	4.98
Crude Fiber	20.78	30.26	20.94
NDF^8^	38.04	61.19	36.44
ADF^9^	22.75	38.08	23.20
ADL^10^	3.48	9.22	3.79
EE^11^	4.42	2.63	5.09
Ash	7.41	8.24	7.22
Starch	20.07	11.79	19.81
NSC^12^	35.46	19.02	35.82
Ca^13^	0.80	0.41	0.80
P tot^14^	0.40	0.54	0.41
P-ava^15^	0.04	0.00	0.04
MNEFA-II^6^	0.20	0.08	0.21
K^17^	0.62	0.00	0.62
Na^18^	0.07	0.00	0.07
Cl^19^	0.11	0.00	0.11

^1^Dry matter; ^2^Unité fourragère lait; ^3^crude protein; ^4^protein digestible; ^5^protein digested in the small intestine when rumen-fermentable nitrogen is limiting; ^6^protein digested in the small intestine when rumen-fermentable energy is limiting; ^7^dietary protein undegraded in the rumen but truly digestible in the small intestine; ^8^neutral detergent fiber; ^9^acid detergent fiber; ^10^acid detergent lignin; ^11^ether extract; ^12^nonstructural carbohydrates; ^13^calcium; ^14^total phosphorous; ^15^available phosphorous; ^16^magnesium; ^17^potassium; ^18^sodium; and ^19^chloride.

### 2.3 Experimental design

A longitudinal experimental design was used, and the animals were evaluated weekly from 7 weeks before the estimated calving date to 7 weeks after postpartum, including the week of parturition, resulting in a total of 14 records for each animal. The timeline included: minus seven (T-7), minus six (T-6), minus five (T-5), minus four (T-4), minus three (T-3), minus two (T-2), and minus one (T-1) weeks before calving; the day of calving (T0); and one (T + 1), two (T + 2), three (T + 3), four (T + 4), five (T + 5), and six (T + 6) weeks after calving. At each time point, blood samples were collected from the jugular vein into Vacutainer serum tubes containing Clot Activator (10 mL, BD Vacutainer®, Becton Dickinson Italia S.p.A., Milano, Italia) using a vacutainer system for each enrolled animal (Supplementary Figure 1). The tubes were refrigerated at 4°C for 30 min and immediately centrifuged at 800 *g* × 10 min (Hettich GmbH & Co., Tuttlingen, Germany). The obtained serum was stocked in an Eppendorf of 1.5 mL at −80°C until biochemical analysis.

### 2.4 Blood analysis

#### 2.4.1 Biochemical analysis

The biochemical analysis was performed using an automatic clinical chemistry analyzer (BS-300; Mindray Bio-medical Electronics Co., Ltd., Shenzhen, China) for the quantification of non-esterified fatty acids (NEFAs; mEq/L); β-hydroxybutyrate (BHB; mmol/L); glucose (GLU; mg/dL); total cholesterol (CHO; mg/dL); triglycerides (TRI; mg/dL); aspartate amino-transferase (AST; UI/L); alanine amino-transferase (ALT; UI/L); alkaline phosphatase (ALP; UI/L); γ-glutamyl-transferase (GGT; UI/L); lactate dehydrogenase (LDH; UI/L); total bilirubin (TBIL; mg/dL); conjugated bilirubin (BC; mg/dL); creatinine (CREA; mg/dL); uric acid (mg/dL); urea (mg/dL); creatine kinase (CK; UI/L); total protein (TP; g/dL); amylase (AMY; UI/L); calcium (Ca; mg/dL); iron (Fe; μg/dL); copper (Cu; μg/dL); phosphorous (P; mg/dL); potassium (K; mmol/L); and magnesium (Mg; mg/dL). The albumin (ALB; g/dL), α-globulin (α-GL; g/dL), β-globulin (β-GL; g/dL), and γ-globulin (γ-GL; g/dL) were evaluated using capillary electrophoresis (Minicap; Sebia Italia S.r.l., Firenze, Italy).

#### 2.4.2 Oxidative stress profiles

The determination of the biological antioxidant potential (BAP) was carried out using commercial kits (BAP Test; Diacron International S.r.l., Grosseto, Italy), and the results were expressed in micromoles per liter (μmol/L) of antioxidants. In contrast, the reactive oxygen metabolites-derived compounds (D.ROMS), mainly hydroperoxides (ROOH), found in serum samples, were measured using a colorimetric kit (d-ROMs Test; Diacron International S.r.l., Grosseto, Italy), and the results from this test were expressed in Carratelli Units (U.CARR), with one U.CARR corresponds to 0.08 mg of hydrogen peroxide (H_2_O_2_) per deciliter of serum.

#### 2.4.3 Acute phase proteins and bactericidal activity of serum

The concentration of haptoglobin (Hp; mg/mL) in the serum was measured by a colorimetric method using the Phase Haptoglobin Colorimetric Assay kit (Tridelta Development Ltd., Kildare, Ireland). Additionally, the lytic activity of the serum was evaluated against 3% rabbit blood cells in veronal buffer to evaluate the hemolytic complement activity (COM; UE/150 mL). For this analysis, samples were appropriately diluted and treated with the hemolytic system and then incubated at 37°C in a humidified thermostat for 30 min. Post-incubation, the samples were centrifuged to separate the supernatant, and the absorbance of this supernatant was measured at 540 nm. The content of 50% hemolytic units in the sample was then determined using a mathematical formula inserted in an electronic spreadsheet (18).

To determine lysozyme concentration (LYS; μg/mL) in serum, an assay involving *Micrococcus Lysodeikticus* embedded in an agar gel was used. Since the microorganism is particularly sensitive to the lytic activity of lysozyme, the lysis halos of the microorganism around the sample deposition were measured. The concentration of lysozyme was calculated based on a standard curve, which was established by incubating the known amounts of lysozyme (18).

The estimation of bactericidal activity of the serum was carried out by using a 96-well microplate method. A known quantity of *E. coli* was cultured in the presence of the test serum, simple broth, and veronal buffer. By spectrophotometric reading of the plates thus prepared, the optical density of the culture wells was measured both in the presence and in the absence of the serum under examination to evaluate the turbidity, a parameter proportional to the bactericidal capacity of the serum (18). The result of this test is expressed as a percentage, indicating the bactericidal capacity of the serum.

### 2.5 Group division

Group division was performed retrospectively according to the NEFA concentration measured at the beginning of the trial (7 weeks before parturition; T-7). The animals were then classified into three groups: NEFA-I or mild lipomobilization group (NEFA≤0.29 mEq/L; 18 animals); NEFA-II or medium mobilization group (0.29 < NEFA<0.57 mEq/L; 20 animals); and NEFA-III or severe lipomobilization group (NEFA≥0.57 mEq/L; 38 animals) (13).

### 2.6 Statistical analysis

The statistical analysis was performed using the S.A.S. system software (version 9.4; SAS Institute Inc., Cary, North Carolina, United States). The normal distributions of biochemical parameters were assessed using the Shapiro–Wilk normality test. A two-way repeated measures ANOVA with the effects of group and time was performed to evaluate the statistical difference between groups and changes over time. A *post hoc* pairwise comparison among least squares means was performed using the Bonferroni correction to adjust for multiple testing. In general, a *p value* of ≤0.05 was considered statistically significant, whereas a 0.05 < *p value* ≤ 0.10 was regarded as indicating a trend toward significance.

## 3 Results

### 3.1 General characteristics

Each group in the study included both primiparous (six in NEFA-I, five in NEFA-II, and 11 in NEFA-III) and multiparous animals (12 in NEFA-I, 15 in NEFA-II, and 27 in NEFA-III). The primiparous animals represented 33, 25, and 29% of the groups NEFA-I, NEFA-II, and NEFA-III, respectively. The BCS decreased from an average value of 7.33 ± 0.12 at T-7 to 7.03 ± 0.12 at T + 6. No statistical differences were found in parity (*p* = 1.000) or BCS (*p* = 0.664) over time among the groups, indicating consistent parity and body condition across the study period.

### 3.2 Biochemical profiles

Among the evaluated biochemical parameters, the following results did not present significant differences over time among the groups (*p* > 0.05): AST, ALT, ALP, GGT, LDH, CK, TBIL, BC, α-GL, TRI, CREA, urea, AMY, Ca, P, Cu, and bactericidal activity. All the mean values and their standard error of the mean are shown in Supplementary Table 1.

### 3.3 Fat mobilization profiles

**Figure 1:**
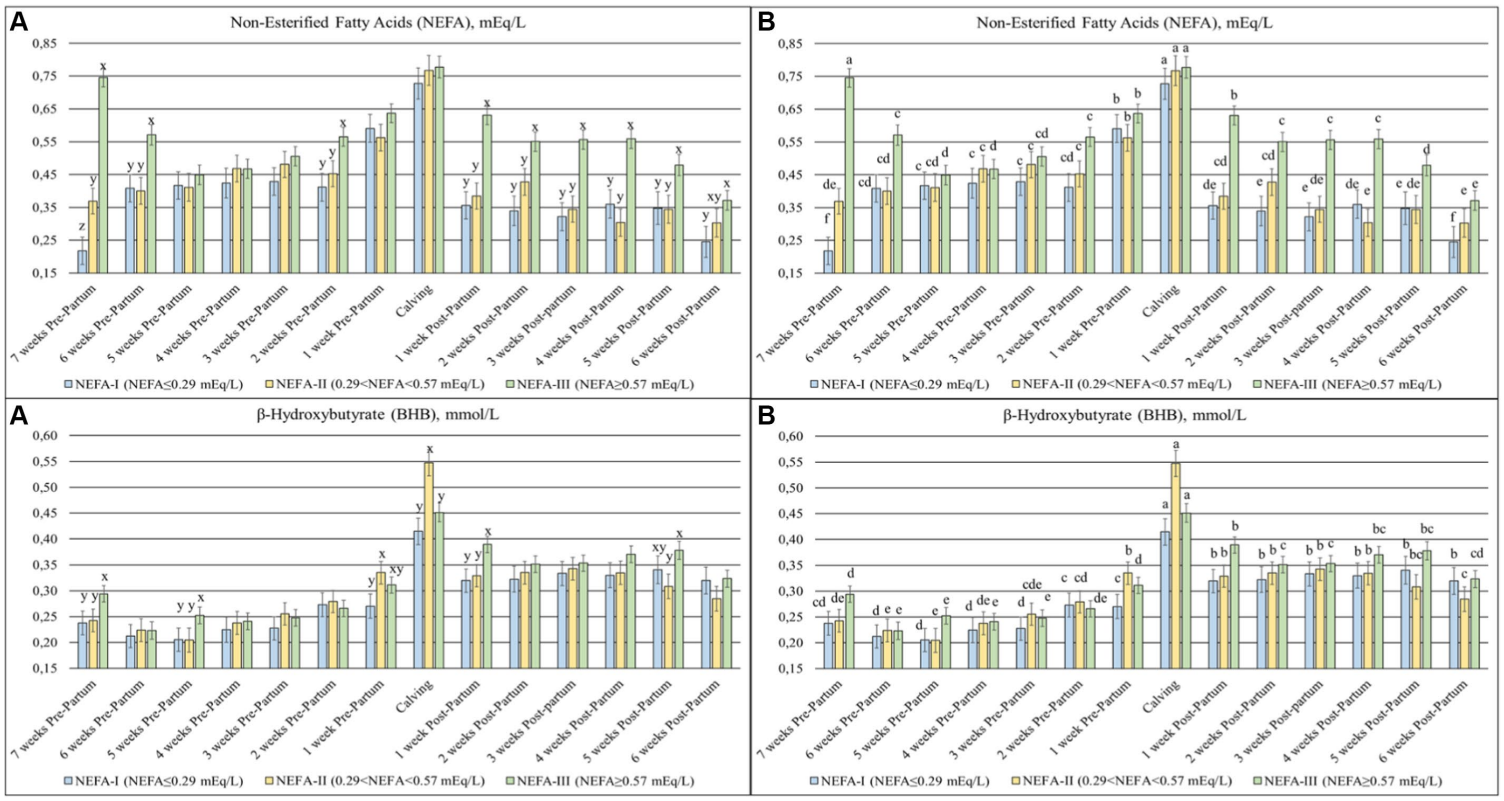
Concentration of non-esterified fatty acids (NEFAs; mEq/L) and β-hydroxybutyrate (BHB; mmol/L), from the prepartum to postpartum periods in dairy buffaloes. **(A)** Significant differences among groups within the same time (^x–z^); **(B)** Significant differences over time within the same group (^a–i^). NEFA-I, *n* = 18: NEFA < 0.29 mEq/L; NEFA-II, *n* = 20: 0.29 mEq/L ≤ NEFA < 0.57 mEq/L; NEFA-III, *n* = 38: NEFA ≥ 0.57 mEq/L; T-7: 7 weeks before calving; T-6: 6 weeks before calving; T-5: 5 weeks before calving; T-4: 4 weeks before calving; T-3: 3 weeks before calving; T-2: 2 weeks before calving; T-1: 1 week before calving; T0: calving; T + 1: 1 week after calving; T + 2: 2 weeks after calving; T + 3: 3 weeks after calving; T + 4: 4 weeks after calving; T + 5: 5 weeks after calving; and T + 6: 6 weeks after calving.

**Figure 2:**
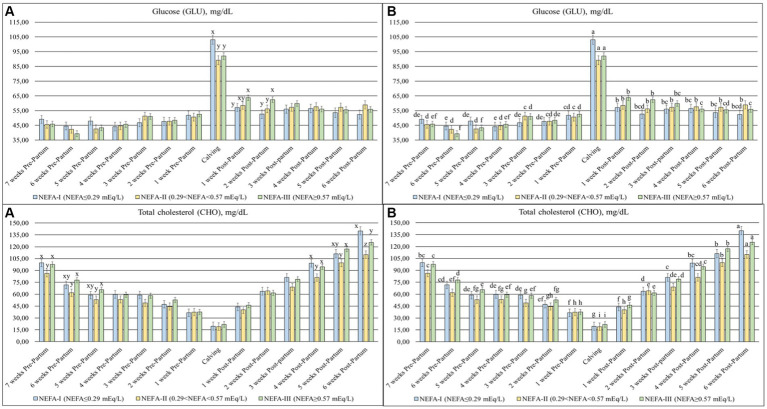
Concentration of glucose (GLU; mg/dL) and total cholesterol (CHO; mg/dL) from the prepartum to postpartum periods in dairy buffaloes. **(A)** Significant differences among groups within the same time (^x–z^); **(B)** Significant differences over time within the same group (^a–i^). NEFA-I, *n* = 18: NEFA<0.29 mEq/L; NEFA-II, *n* = 20: 0.29 mEq/L ≤ NEFA < 0.57 mEq/L; NEFA-III, *n* = 38: NEFA ≥ 0.57 mEq/L; T-7: 7 weeks before calving; T-6: 6 weeks before calving; T-5: 5 weeks before calving; T-4: 4 weeks before calving; T-3: 3 weeks before calving; T-2: 2 weeks before calving; T-1: 1 week before calving; T0: calving; T + 1: 1 week after calving; T + 2: 2 weeks after calving; T + 3: 3 weeks after calving; T + 4: 4 weeks after calving; T + 5: 5 weeks after calving; and T + 6: 6 weeks after calving.

Statistical differences were found in NEFA (*p* < 0.001), BHB (*p* = 0.047) (Figure 1), GLU (*p* = 0.084), and CHO (*p* = 0.017) (Figure 2) among the groups and over time. Slight differences were identified in the NEFA concentration during the prepartum period, despite the initial greatest value in the NEFA-III and a greater value in the NEFA-II. All groups showed a progressive increment in this parameter toward calving (T0), followed by a decrease during the postpartum period. However, the NEFA-III showed the greatest concentration during the early lactation. Both BHB and GLU concentrations showed a peak at calving (T0) and greater levels during the postpartum period than the prepartum period. The greatest level of BHB at T0 was evidenced in the NEFA-II as during the previous week (T-1; 1-week before calving), while the greatest concentrations during the postpartum period were evidenced in the NEFA-III at 1 and 5 weeks after calving (T + 1 and T + 5). The group NEFA-I showed the greatest peak at calving (T0) of GLU, whereas the greatest level of GLU was present in the NEFA-III at 1 and 2 weeks after calving (T + 1 and T + 2). The total cholesterol concentration reduced around calving (T0), where it reached its nadir. The NEFA-II showed the lowest CHO concentration during the first 3 weeks of the trial (7, 6, and 5 weeks before calving or T-7, T-6, and T-5) and the last 3 weeks (4, 5, and 6 weeks after calving or T + 4, T + 5, and T + 6).

### 3.4 Oxidative stress profiles and uric acid

**Figure 3:**
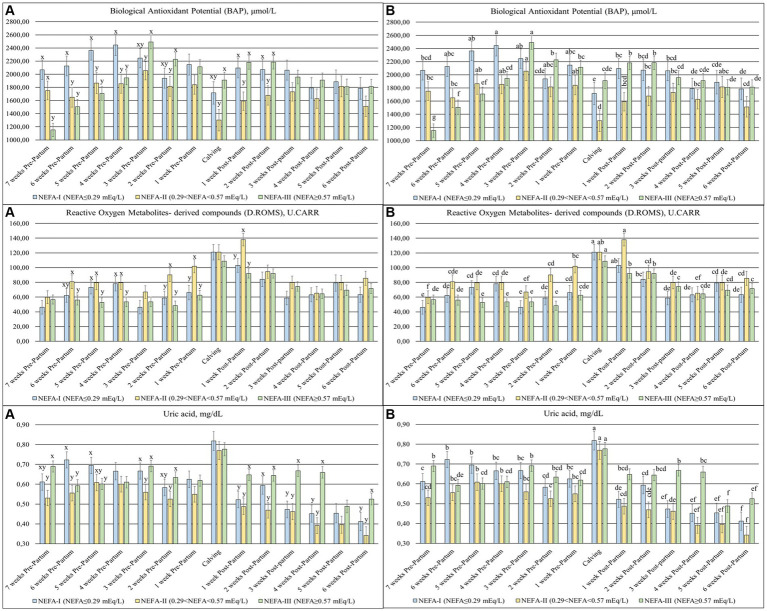
Concentration of biological antioxidant potential (BAP; ×10^2^ mol/L), reactive oxygen metabolites—derived compound (D.ROMS; U.CARR), and uric acid (mg/dL) from the prepartum to postpartum periods in dairy buffaloes. (**A**) Significant differences among groups within the same time (^x–z^); (**B**) Significant differences over time within the same group (^a–i^). NEFA-I, *n* = 18: NEFA < 0.29 mEq/L; NEFA-II, *n* = 20: 0.29 mEq/L ≤ NEFA<0.57 mEq/L; NEFA-III, *n* = 38: NEFA ≥ 0.57 mEq/L; T-7: 7 weeks before calving; T-6: 6 weeks before calving; T-5: 5 weeks before calving; T-4: 4 weeks before calving; T-3: 3 weeks before calving; T-2: 2 weeks before calving; T-1: 1 week before calving; T0: calving; T + 1: 1 week after calving; T + 2: 2 weeks after calving; T + 3: 3 weeks after calving; T + 4: 4 weeks after calving; T + 5: 5 weeks after calving; T + 6: 6 weeks after calving.

The BAP (*p* < 0.001), D. ROMS (*p* = 0.080) (Figure 3), and uric acid (*p* < 0.001) were significantly different among the groups over time. The BAP concentrations were highly variable during the study period, with a decrease at calving (T0), while the D. ROMS showed a peak between calving (T0) and 1 week after calving (T + 1). The first 4 weeks of the trial (T-7 to T-4) showed the greatest availability of BAP in the NEFA-I and the lowest value generally in the NEFA-III. In the same time frame, the greatest D. ROMS was evidenced in the NEFA-I and the NEFA-II, and the lowest value was evidenced in the NEFA-III. From 3 weeks before to 2 weeks after calving (T-3 to T + 2), the greatest BAPs were generally shown in the NEFA-III, while the lowest values were found in the NEFA-II. This group, NEFA-II, showed the greatest level of D. ROMS from 2 weeks before to 1 week after calving (T-2 to T + 1). The uric acid decreased over the early lactation, especially in the NEFA-I and NEFA-II. The lowest concentration in both the prepartum and postpartum periods was generally found in the NEFA-II, while the highest values were generally found in the NEFA-I during the prepartum period and the NEFA-III during the postpartum period.

### 3.5 Protein fraction

**Figure 4:**
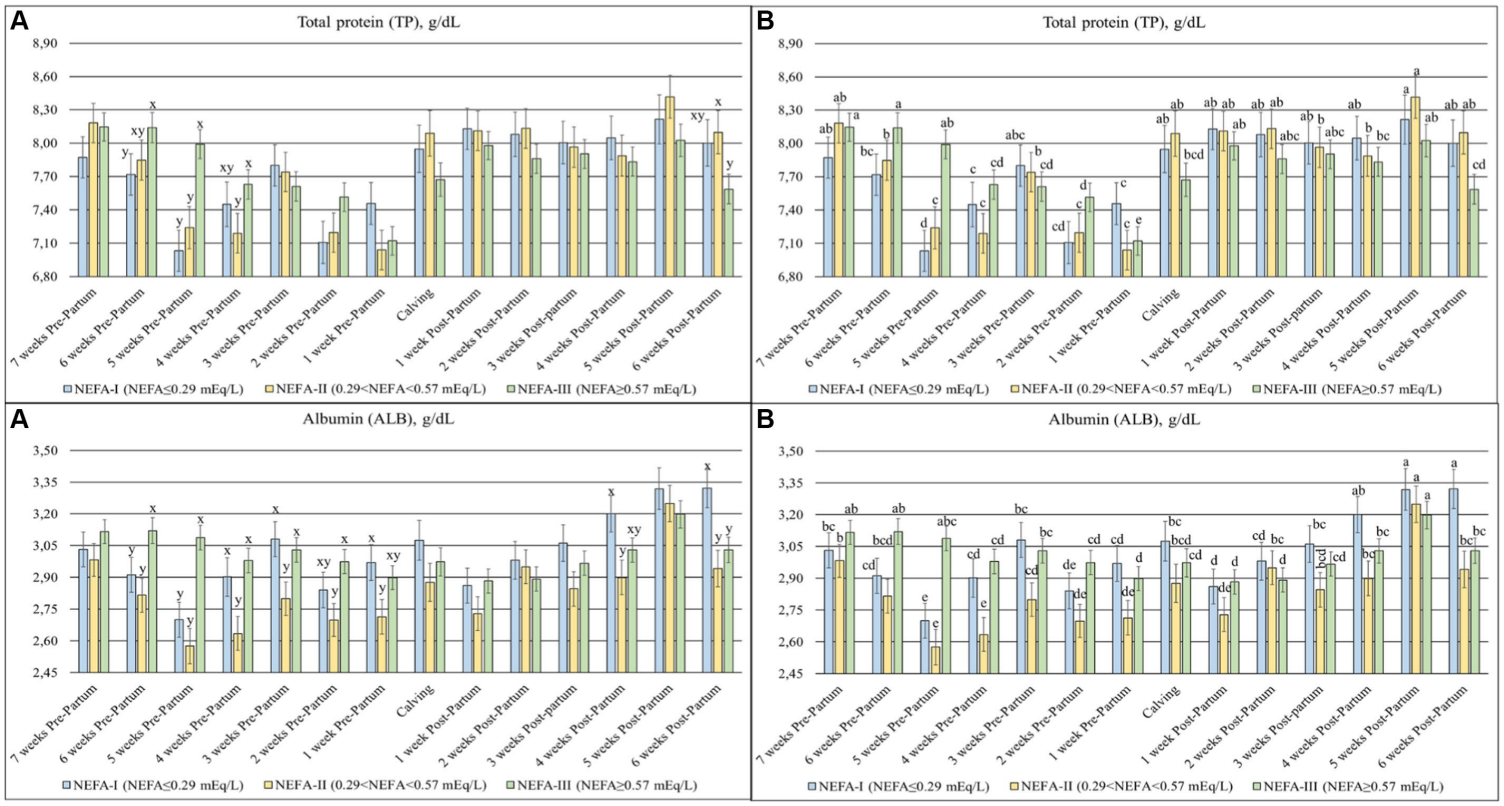
Concentration of total protein (TP; g/dL) and albumin (ALB; g/dL) from the prepartum to postpartum periods in dairy buffaloes. (**A**) Significant differences among groups within the same time (^x–z^); (**B**) Significant differences over time within the same group (^a–i^). NEFA-I, *n* = 18: NEFA < 0.29 mEq/L; NEFA-II, *n* = 20: 0.29 mEq/L ≤ NEFA < 0.57 mEq/L; NEFA-III, *n* = 38: NEFA ≥ 0.57 mEq/L; T-7: 7 weeks before calving; T-6: 6 weeks before calving; T-5: 5 weeks before calving; T-4: 4 weeks before calving; T-3: 3 weeks before calving; T-2: 2 weeks before calving; T-1: 1 week before calving; T0: calving; T + 1: 1 week after calving; T + 2: 2 weeks after calving; T + 3: 3 weeks after calving; T + 4: 4 weeks after calving; T + 5: 5 weeks after calving; T + 6: 6 weeks after calving.

**Figure 5:**
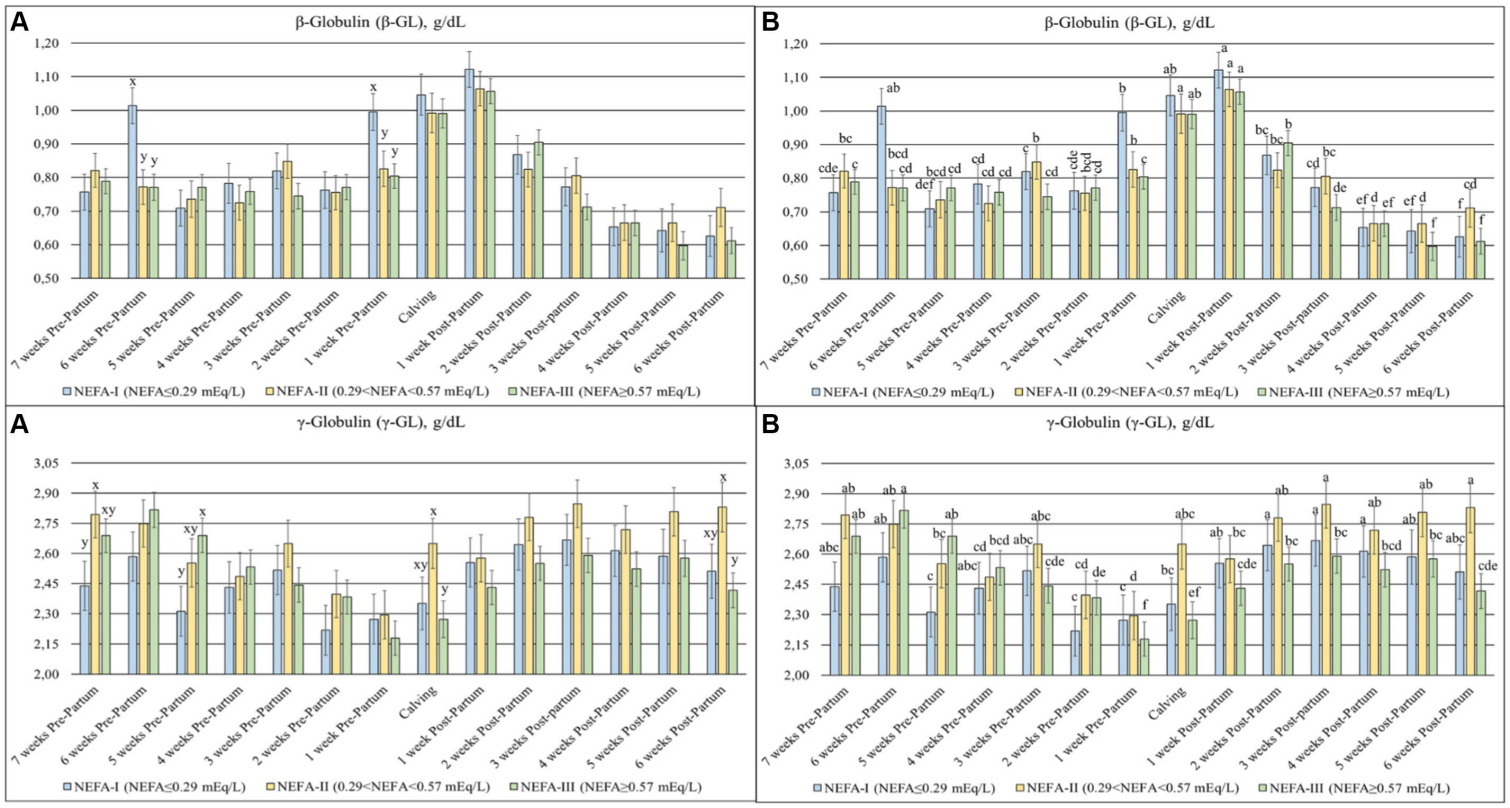
Concentration of β-globulin (β-GL; g/dL) and γ-globulin (γ-GL; g/dL) from the prepartum to postpartum periods in dairy buffaloes. (**A**) Significant differences among groups within the same time (^x–z^); (**B**) Significant differences over time within the same group (^a–i^). NEFA-I, *n* = 18: NEFA < 0.29 mEq/L; NEFA-II, *n* = 20: 0.29 mEq/L ≤ NEFA < 0.57 mEq/L; NEFA-III, *n* = 38: NEFA ≥ 0.57 mEq/L; T-7: 7 weeks before calving; T-6: 6 weeks before calving; T-5: 5 weeks before calving; T-4: 4 weeks before calving; T-3: 3 weeks before calving; T-2: 2 weeks before calving; T-1: 1 week before calving; T0: calving; T + 1: 1 week after calving; T + 2: 2 weeks after calving; T + 3: 3 weeks after calving; T + 4: 4 weeks after calving; T + 5: 5 weeks after calving; T + 6: 6 weeks after calving.

The TP (*p* = 0.003), ALB (*p* = 0.007) (Figure 4), β-GL (*p* = 0.012), and γ-GL (*p* = 0.058) (Figure 5) showed a statistical interaction between group and time. The TP showed a slight variation with a general tendency to decrease toward calving during the prepartum period. On the contrary, this parameter was more stable, with greater values obtained during the postpartum period. The group NEFA-III had the greatest values between 6 and 4 weeks before calving (T-6 to T-4). The levels of ALB varied during the trial, with a tendency to increase at the end of the study period. The highest concentrations were observed in the NEFA-III during the prepartum period, while the lowest concentrations were evidenced in the NEFA-II. Furthermore, the group NEFA-I showed the highest levels of ALB at 4 and 6 weeks after calving (T + 4 and T + 6). Around the time of calving, there were noticeable fluctuations in other biochemical markers: β-glutamyltransferase (β-GL) levels peaked at 1 week after calving (T + 1), indicating heightened liver activity, while γ-glutamyltransferase (γ-GL) levels were the lowest at 1 week before calving (T-1), suggesting variations in metabolic stress or hepatic function during these critical periods. Only two specific time points showed significant differences among the groups in β-GL levels: 6 and 1 weeks before calving (T-6 and T-1), with the greatest concentration in the NEFA-I. The lowest levels of γ-GL were shown in the NEFA-I during the prepartum period, specifically at 7 and 5 weeks before calving (T-7 and T-5), whereas the greatest concentration was shown in the NEFA-II or the NEFA-III. On the contrary, the highest level was evidenced in the NEFA-II, and the lowest level was evidenced in the NEFA-III at the time of calving (T0) and 6 weeks after calving (T + 6).

### 3.6 Acute phase proteins

**Figure 6:**
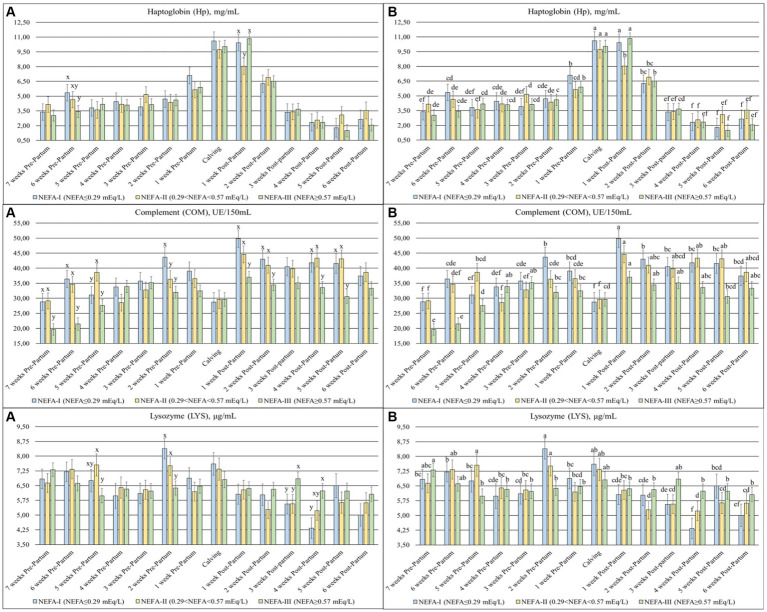
Concentration of haptoglobin (Hp; mg/mL), complement (COM; UE/150 mL), and lysozyme (LYS; μg/mL) from the prepartum to postpartum periods in dairy buffaloes. (**A**) Significant differences among groups within the same time (^x–z^); (**B**) Significant differences over time within the same group (^a–i^). NEFA-I, *n* = 18: NEFA < 0.29 mEq/L; NEFA-II, *n* = 20: 0.29 mEq/L ≤ NEFA<0.57 mEq/L; NEFA-III, *n* = 38: NEFA≥0.57 mEq/L; T-7: 7 weeks before calving; T-6: 6 weeks before calving; T-5: 5 weeks before calving; T-4: 4 weeks before calving; T-3: 3 weeks before calving; T-2: 2 weeks before calving; T-1: 1 week before calving; T0: calving; T + 1: 1 week after calving; T + 2: 2 weeks after calving; T + 3: 3 weeks after calving; T + 4: 4 weeks after calving; T + 5: 5 weeks after calving; T + 6: 6 weeks after calving.

The Hp (*p* = 0.059), COM (*p* = 0.002), and LYS (*p* = 0.020) (Figure 6) were significantly different among the groups over time. The Hp showed a peak between calving (T0) and after the first week (T + 1), with the lowest value in the group NEFA-II at T + 1. The COM was generally higher in the postpartum than the prepartum, with the lowest values in the NEFA-III for both periods. On the other hand, the LYS was generally greater in the prepartum than in the postpartum period. The group NEFA-III showed the lowest values in prepartum, specifically at 5 and 2 weeks before calving (T-5 and T-2), whereas the same group showed the highest value in the postpartum period, specifically at 3 and 4 weeks after calving (T + 3 and T + 4).

### 3.7 Mineral profile

**Figure 7:**
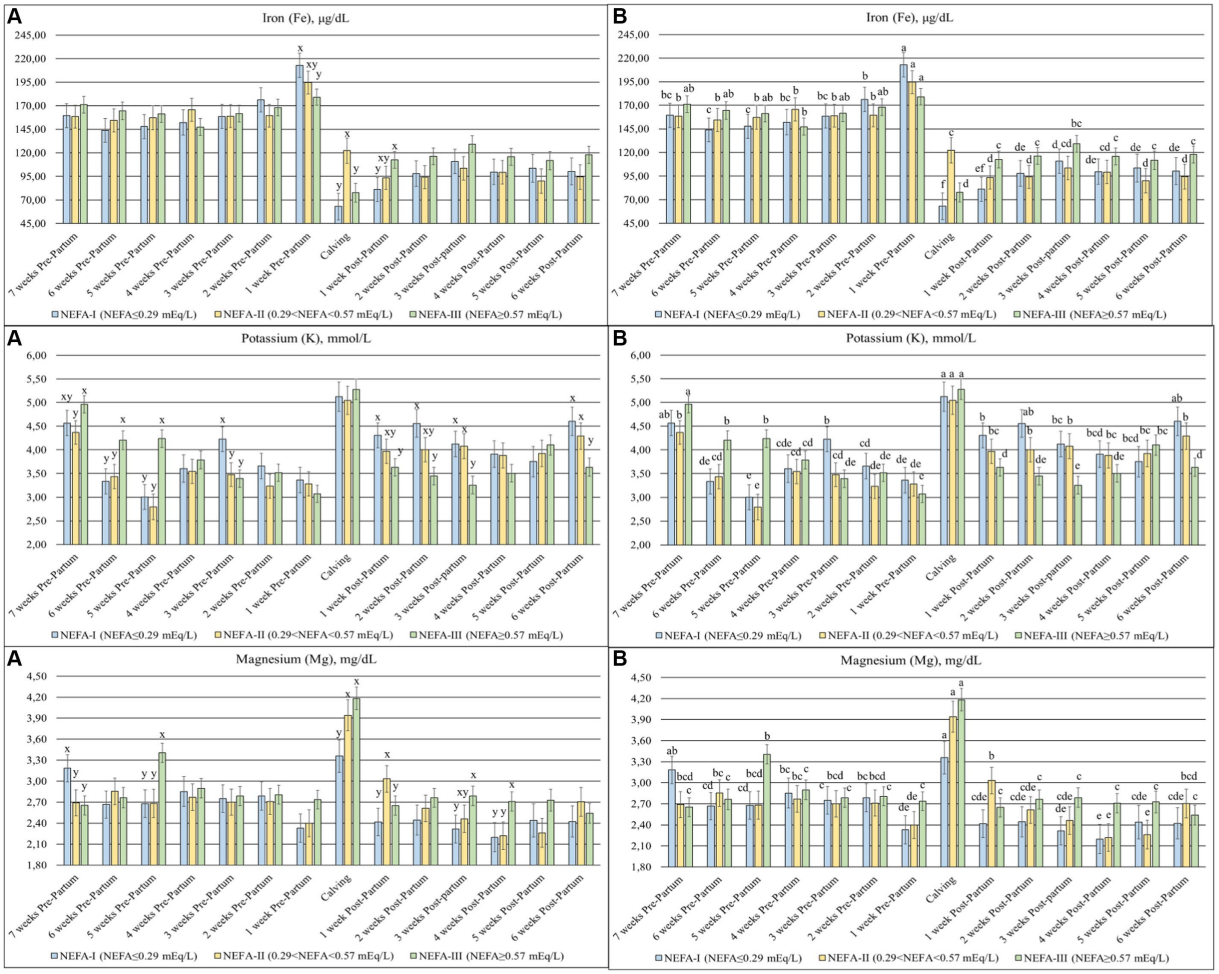
Concentration of iron (Fe; μg/dL), potassium (K; mmol/L), and magnesium (Mg; mg/dL) from the prepartum to postpartum periods in dairy buffaloes. (**A**) Significant differences among groups within the same time (^x–z^); (**B**) Significant differences over time within the same group (^a–i^). NEFA-I, *n* = 18: NEFA<0.29 mEq/L; NEFA-II, *n* = 20: 0.29 mEq/L ≤ NEFA < 0.57 mEq/L; NEFA-III, *n* = 38: NEFA ≥ 0.57 mEq/L; T-7: 7 weeks before calving; T-6: 6 weeks before calving; T-5: 5 weeks before calving; T-4: 4 weeks before calving; T-3: 3 weeks before calving; T-2: 2 weeks before calving; T-1: 1 week before calving; T0: calving; T + 1: 1 week after calving; T + 2: 2 weeks after calving; T + 3: 3 weeks after calving; T + 4: 4 weeks after calving; T + 5: 5 weeks after calving; T + 6: 6 weeks after calving.

Among minerals, Fe (*p* = 0.078), K (*p* < 0.001), and Mg (*p* = 0.051) changed among the groups over time (Figure 7). The Fe levels were higher in the prepartum period than in the postpartum period, with a peak 1 week before calving (T-1). The NEFA-I showed the highest peak at T-1, while the same group showed the lowest value at calving (T0) and after 1 week (T + 1). The K decreased over the prepartum period, with a peak at calving (T0). During this period, the greatest levels were found, especially in the NEFA-III, whereas the same group generally showed the lowest levels during the postpartum period. Mg showed a peak of concentration at calving (T0), with the lowest peak in the NEFA-I. In early lactation, the NEFA-II showed the highest concentration at 1 week after calving (T + 1), while the NEFA-III showed the highest concentration at 3 and 4 weeks after calving (T + 3 and T + 4).

## 4 Discussion

In accordance with the principles of the 3Rs (replacement, reduction, and refinement), the choice to conduct the experiment on a single farm was influenced by the desire to minimize the number of animals used, while ensuring robust results, an objective made possible by the homogeneity of the experimental conditions. The farm under study allowed us to have only one exposure scenario, especially in terms of the type of feed and rationing, which allowed for the execution of accurate metabolic profiles on an appropriate number of subjects. To date, studies on the metabolic aspects of the buffalo species have been conducted either using minimal numbers of animals or constructing curves through single sampling on multiple subjects, reconstructing possible profiles through interpolations. In this study, we preferred to construct real profile curves with a considerable expenditure of energy and funds considering a total number of 1,064 samples and 36,176 analyses.

The negative energy balance naturally occurs from late gestation to early lactation in ruminants, although buffaloes seem to show a different trend than dairy cows (19, 20). One of the metabolic adaptations is adipose tissue lipolysis, resulting in an increase in the NEFA (21, 22). The release of the NEFA is also related to the formation of ketone bodies, particularly BHB, which can negatively influence animal health by causing metabolic disorders (5). Typically, the NEFA peak is followed by the BHB peak, but large variations in the timing of the peak can occur (23). This study aimed to evaluate the biochemical profile from the prepartum to postpartum periods in Mediterranean buffaloes with mild (NEFA-I; NEFA≤0.29 mEq/L), medium (NEFA-II; 0.29 < NEFA<0.57 mEq/L), and severe (NEFA-III; NEFA≥0.57 mEq/L) lipomobilization at 7 weeks before calving (T-7).

The pattern of NEFA and BHB concentrations suggested that the NEFA-III group exhibited more energy deficiency than other groups during the dry-off period, resulting in increased lipid mobilization, although the same TMR was used for all animals. In addition, the NEFA-II group showed lipolytic activity indicative of energy deficiency, although less evident than the NEFA-III group. As shown in the studies of Fiore et al. (19) and Deka et al. (21) on dairy buffaloes, all groups showed a peak in NEFA and BHB near calving, probably due to the smaller abdominal space resulting in reduced voluntary dry matter intake and increased energy requirements for calving and the onset of lactation (21, 24). However, the NEFA-III group continued to show the highest NEFA concentration during the postpartum period. Furthermore, BHB showed the highest values in the same group at 1 and 5 weeks after calving (T + 1 and T + 5). This finding is in line with the trend of BHB in dairy cows. In fact, the highest percentage of hyperketonemia cases occurs before the 10th day of lactation, with a second increase occurring between the 1st and the 2nd month of lactation (25). Buffaloes generally show a peak in milk production between 20 and 30 days postpartum, with a consequent increase in energy demand (19). However, an increase in BHB may not only be related to an impairment of Krebs cycle function but also to an energy deficiency (26). Therefore, the NEFA and BHB results of the NEFA-III group suggested the persistence of energy deficits during the postpartum period. In addition, the higher BHB levels at 1 and 5 weeks postpartum could indicate both increased metabolic stress from greater lipomobilization and a potential Krebs cycle impairment.

The lower GLU concentration in the NEFA-II and NEFA-III groups at calving probably causes higher lipomobilization and the formation of BHB. Furthermore, the association of this result with a possible impaired Krebs cycle function could have caused the significant BHB peak in the NEFA-II group. Moreover, GLU showed the highest concentration in the NEFA-III group at 1 and 2 weeks after calving (T + 1 and T + 2) despite the highest lipomobilization during the same period. This result could support the hypothesis that GLU concentrations are poorly modified parameters during the initial conditions of metabolomic alterations (26).

The total cholesterol concentration decreased during the prepartum period of the ruminants and then increased subsequently after calving, as shown in this study (19, 27). The decrease in CHO may be associated with steroidogenesis for the production of estrogens, progesterone, and glucocorticoids during the last period of pregnancy (19, 28). Furthermore, this decrease may represent a limiting factor for lipoprotein synthesis, particularly for very low-density lipoprotein (VLDL) composed of cholesterol esters (29, 30). Cholesterol esters are derived from the activity of lecithin cholesterol acyltransferase (LCAT), whose functionality is reduced before and shortly after calving. The reduced levels of CHO potentially associated with reduced activity of LCAT may predispose animals to fatty liver disease (30–32). The NEFA-II group showed the lowest concentration during the first 3 weeks of the study (T-7, T-6, and T-5) and in the last 3 weeks (T + 4, T + 5, and T + 6), further predisposing this group to fatty liver disease.

The production of free radicals as reactive oxygen species (ROS) is particularly increased during the transition period in animals due to increased metabolic demands and lipid peroxidation. The excess of radicals over antioxidants induces oxidative stress that increases the susceptibility of animals in the transition period to various production diseases (20, 33). The NEFA-III group in this study started with the lowest concentration of antioxidants (BAP), which increased during the prepartum period, which is in agreement with the lowest concentration of radicals (D.ROMS). Uric acid is one of the antioxidants considered in the BAP test (33). Its concentration was the highest at the beginning of the study in the group NEFA-III, in contrast to the BAP levels, and remained elevated during the postpartum period. The increase in uric acid in dairy cows is considered a response to oxidative stress resulting from inflammation (34). In contrast, the NEFA-II group showed lower concentrations of antioxidants, uric acid included, during the prepartum period, with the highest concentration of radicals. These results suggest a higher risk of oxidative stress for the NEFA-II group.

Considering the above results and the absence of differences in both the BCS and the diet of the animals, it can be assumed that the NEFA-III group was able to manage lipomobilization without exacerbating oxidative stress during both the prepartum and postpartum periods. On the other hand, the NEFA-II group was not fully able to manage lipomobilization, resulting in an increased risk of oxidative stress during the prepartum period. These conditions may suggest a possible genetic and/or metabolic deficiency of the NEFA-II and NEFA-III groups that could increase the risk of metabolic diseases.

Inflammatory events may occur around parturition due to differentiation and proliferation of the mammary gland, the interaction between the uterus and placenta, and physical effort during calving (29, 35). Serum proteins are divided into ALB, α, β, and γ–GL (36). A decrease in TP levels during the dry-off period of buffaloes, as observed in this study, up to the onset of lactation has previously been shown, probably due to hormone synthesis, fetal growth, especially for muscle masses, and gluconeogenesis (20, 37). On the contrary, ALB is a negative acute-phase protein whose concentration should remain constant during pregnancy in healthy cows (38). The NEFA-II group showed the lowest levels of TP and ALB during the prepartum period, suggesting an inflammatory status probably related to the highest values of radicals during the same period. An inflammatory condition should also be characterized by a higher concentration of GL, especially in α–GL, as it is more composed of positive acute phase proteins (Hp, ceruloplasmin, α1-acid glycoprotein, and α1-antitrypsin) (35, 36). In our study, α–GL and Hp showed no differences among the groups during the prepartum period.

A slight increase in Hp can be evidenced, especially during the last weeks of pregnancy, as demonstrated in this study, coinciding with the mammary gland development (35). Inflammatory events may also be inferred by an increase in β-GL levels, as found in ketotic cows (39–41). Furthermore, a decrease in γ–GL is observed in dairy cows during the last month of pregnancy until the first days of lactation due to the transfer of immunoglobulins from the blood into the colostrum (39, 42, 43). The buffaloes in this study showed an increase in β-GL and a decrease in γ–GL at or around the time of calving across all groups.

Lysozyme (LYS) is an antibacterial enzyme produced by neutrophils and macrophages and is present in all body fluids. Along with the complement system (COM), LYS plays an important role in the innate defense against pathogens. Typically, LYS concentrations are usually higher in buffaloes than in cows (17–20 vs. 1–3 μg/mL) (44, 45). A lower LYS level is indicative of inflammation and is associated with a decrease in COM levels (35). In our study, the NEFA-III group showed lower levels of both LYS and COM during the prepartum period. After calving, COM levels remained lower in this group compared to others, while LYS levels increased. Furthermore, LYS concentrations across all groups were generally lower at all time points compared to previously reported values for the buffaloes (44). Similarly, COM levels during the first three weeks (T-7, T-6, and T-5) of the study in the NEFA-III group were lower compared to previous findings (45). These results suggest that all groups experienced an inflammatory state close to calving. However, the NEFA-III group may be more susceptible to inflammation, considering their lower levels of γ–GL, LYS, and COM and higher levels of NEFA (35).

Iron is an important component of proteins involved in oxygen transport and energy production (46). Its level usually ranges between 130 and 250 μg/dL in ruminants (47), although according to Rocha et al. (48), its concentration can range between 93 and 287 μg/dL in buffaloes. Our animals were within the normal range, whereas an increase was observed in all the groups at T-1. Increased iron levels may be related to causing liver disease (47). NEFA values suggest that lipomobilization is close to calving across all groups, which could have a negative impact on liver health. Interestingly, the NEFA-III group showed the highest values among groups at 1 week after calving. In contrast, the lower Fe levels evidenced during the postpartum period compared to the prepartum period may be due to inflammatory events, which are normal during the peripartum period (47). Regarding K and Mg, all groups showed an increase in the concentration of both minerals at the time of calving. Considering that K is predominantly intracellular, the increase may suggest an intensification of metabolically active body tissue and muscle, probably due to calving itself (49). Furthermore, the NEFA-III group showed the highest values during the first 3 weeks of the study. On the contrary, the increase in Mg could be related to an enhanced requirement for milk production with a consequent increase in bone mobilization (50).

The results of the present study suggest that further refinements in buffalo-specific nutrition and metabolism are needed. Indeed, animals with severe lipomobilization did not show signs of oxidative stress, unlike those with medium lipomobilization. In recent years, it has been hypothesized that certain nutritional requirements, particularly relative to amino acids, may be related to inflammatory, immune, and antioxidant system responses, as well as to the proper functioning of metabolism itself (9, 15, 51). Therefore, insights with more accurate analytical techniques, such as metabolomics, could be useful for identifying and assessing potential nutritional deficiencies and/or increased requirements in these animals. This, in turn, could impact their metabolism, inflammatory, immune, and antioxidant responses. Furthermore, greater lipomobilization could adversely affect and/or be associated with a reduced ability to adequately regulate anti-inflammatory and immune responses. Therefore, animals with high lipomobilization might be more susceptible to infectious diseases (metritis, mastitis, and pneumonia), even in the absence of immunosuppression related to pathological levels of BHB.

## 5 Conclusion

In summary, this study evaluates the biochemical profile of Mediterranean buffaloes with varying levels of lipomobilization during the transition period, from the prepartum to postpartum periods, and describes the trends for the three examined NEFA thresholds. Buffaloes with higher NEFA levels (>0.29 mEq/L; NEFA-II and NEFA-III) 7 weeks before calving should be monitored more closely to mitigate the risk of metabolic diseases. Furthermore, the medium (NEFA-II) and severe (NEFA-III) lipomobilization groups could be associated with differences in the animals’ ability to manage their metabolic status. Specifically, severe lipomobilization was most associated with a greater energy deficit in both prepartum and postpartum periods but without oxidative stress. On the contrary, medium lipomobilization was associated with a less severe energy deficit, accompanied by an inflammatory status and oxidative stress during the prepartum period. These conditions suggest different response mechanisms to metabolic stressors, depending on the ability of buffaloes to maintain homeostasis. Therefore, further studies are needed to investigate the distinctive characteristics of this species in greater depth.

## Data availability statement

The original contributions presented in the study are included in the article/Supplementary material, further inquiries can be directed to the corresponding author.

## Ethics statement

The animal studies were approved by Il protocollo sperimentale dello studio è stato autorizzato dal Ministero della Salute (autorizzazione n. 758/2017-PR). The studies were conducted in accordance with the local legislation and institutional requirements. Written informed consent was obtained from the owners for the participation of their animals in this study.

## Author contributions

AL: Data curation, Writing – original draft, Methodology, Writing – review & editing. EM: Data curation, Methodology, Writing – original draft, Writing – review & editing. GC: Writing – original draft, Data curation, Validation, Writing – review & editing. AM: Investigation, Writing – original draft, Writing – review & editing. BM: Conceptualization, Writing – original draft, Writing – review & editing. JS: Conceptualization, Writing – original draft, Writing – review & editing. GV: Data curation, Writing – original draft, Writing – review & editing. ID: Writing – original draft, Writing – review & editing. LS: Formal Analysis, Writing – original draft, Writing – review & editing. EC: Methodology, Writing – original draft, Writing – review & editing. PR: Data curation, Writing – original draft, Writing – review & editing. BC: Formal Analysis, Writing – original draft, Writing – review & editing. EF: Data curation, Writing – original draft, Writing – review & editing. DV: Funding acquisition, Methodology, Resources, Supervision, Validation, Writing – original draft, Writing – review & editing.
