# Supplementary figures and images for: Biochemical profile differences during the transition period based on different levels of non-esterified fatty acids at 7 weeks before parturition in Mediterranean Italian dairy buffaloes (Bubalus bubalis)
Source: Front Vet Sci. 2024 Jul 2;11:1404041. doi: 10.3389/fvets.2024.1404041 (PMC11250106; doi:10.3389/fvets.2024.1404041)

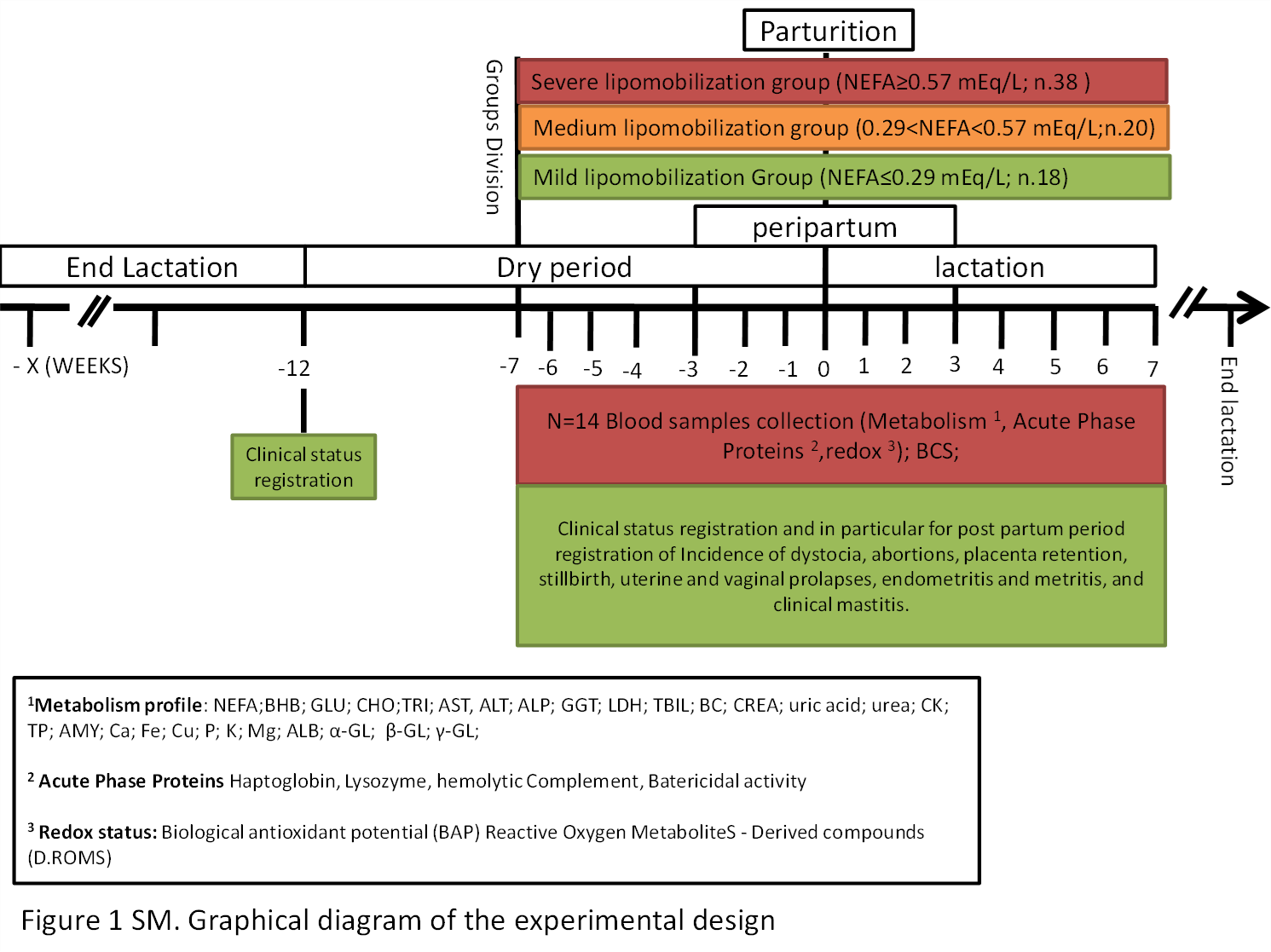

Supplement: Supplementary file 1 [file Image_1.tif]
