# Supplementary material for: Biochemical profile differences during the transition period based on different levels of non-esterified fatty acids at 7 weeks before parturition in Mediterranean Italian dairy buffaloes (Bubalus bubalis)
Source: Front Vet Sci. 2024 Jul 2;11:1404041. doi: 10.3389/fvets.2024.1404041 (PMC11250106; doi:10.3389/fvets.2024.1404041)
Supplement: Supplementary file 2 [file Table_1.DOCX]

Supplementary Material

**Supplementary table 1.** Mean value and standard error of the mean (SEM) from pre-partum to post-partum period for parameters without a significant interaction between groups and time in dairy buffaloes.

|  | ALP (U/L) | | | ALT (U/L) | | | Amylase (U/L) | | | AST (U/L) | | |
| --- | --- | --- | --- | --- | --- | --- | --- | --- | --- | --- | --- | --- |
|  | NEFA-I | NEFA-II | NEFA-III | NEFA-I | NEFA-II | NEFA-III | NEFA-I | NEFA-II | NEFA-III | NEFA-I | NEFA-II | NEFA-III |
| T-7 | 351.4 | 266.8 | 299.8 | 87.3 | 82.8 | 79.1 | 24.6 | 26.1 | 30.4 | 126.1 | 139.3 | 119.0 |
| T-6 | 288.0 | 266.0 | 300.9 | 84.4 | 82.3 | 76.4 | 26.5 | 30.5 | 29.6 | 135.2 | 132.3 | 117.2 |
| T-5 | 290.2 | 238.2 | 282.3 | 84.1 | 81.6 | 81.8 | 23.8 | 30.1 | 31.7 | 126.3 | 132.9 | 121.2 |
| T-4 | 303.4 | 246.8 | 295.3 | 90.8 | 84.2 | 77.4 | 26.3 | 29.4 | 27.6 | 136.0 | 132.4 | 124.8 |
| T-3 | 323.6 | 249.9 | 306.8 | 92.4 | 85.8 | 79.0 | 24.7 | 29.9 | 28.9 | 135.1 | 136.7 | 130.2 |
| T-2 | 288.9 | 270.1 | 292.9 | 83.6 | 79.5 | 77.9 | 23.5 | 28.6 | 29.0 | 127.0 | 130.8 | 130.4 |
| T-1 | 275.9 | 267.0 | 275.6 | 79.2 | 76.1 | 68.7 | 27.3 | 29.3 | 30.3 | 133.6 | 132.3 | 126.4 |
| T0 | 275.6 | 271.1 | 268.1 | 58.3 | 65.4 | 59.7 | 36.3 | 42.4 | 43.7 | 132.0 | 144.6 | 135.7 |
| T+1 | 337.4 | 318.2 | 342.2 | 78.4 | 86.5 | 77.1 | 24.8 | 30.4 | 34.8 | 153.8 | 169.4 | 160.1 |
| T+2 | 328.3 | 335.7 | 343.1 | 86.1 | 94.5 | 83.4 | 23.8 | 31.0 | 31.5 | 146.7 | 162.3 | 156.6 |
| T+3 | 318.4 | 301.5 | 366.7 | 95.1 | 96.3 | 91.2 | 22.8 | 28.9 | 31.0 | 160.3 | 173.2 | 161.7 |
| T+4 | 355.6 | 301.9 | 375.4 | 105.4 | 100.8 | 98.4 | 25.5 | 30.2 | 35.4 | 165.7 | 166.6 | 168.9 |
| T+5 | 355.8 | 376.1 | 427.8 | 117.9 | 108.8 | 106.8 | 25.1 | 29.8 | 35.2 | 169.3 | 171.0 | 171.8 |
| T+6 | 364.9 | 361.6 | 407.9 | 122.3 | 111.4 | 107.6 | 25.4 | 31.9 | 34.1 | 161.7 | 158.5 | 154.1 |
| SEM | 49.6 | 46.8 | 34.0 | 4.82 | 4.54 | 3.29 | 2.47 | 2.32 | 1.68 | 7.25 | 6.81 | 4.94 |
|  | Bactericidial activity (%) | | | Total bilirubin (mg/dL) | | | Conjugated bilirubin (mg/dL) | | | Ca (mg/dL) | | |
|  | NEFA-I | NEFA-II | NEFA-III | NEFA-I | NEFA-II | NEFA-III | NEFA-I | NEFA-II | NEFA-III | NEFA-I | NEFA-II | NEFA-III |
| T-7 | 88.5 | 89.4 | 85.4 | 0.29 | 0.34 | 0.28 | 0.13 | 0.11 | 0.15 | 10.7 | 10.6 | 10.6 |
| T-6 | 91.2 | 91.3 | 80.1 | 0.35 | 0.26 | 0.31 | 0.11 | 0.09 | 0.09 | 10.9 | 10.8 | 10.6 |
| T-5 | 90.1 | 93.3 | 86.4 | 0.31 | 0.28 | 0.28 | 0.09 | 0.08 | 0.08 | 10.6 | 10.2 | 10.9 |
| T-4 | 92.0 | 89.1 | 82.4 | 0.31 | 0.27 | 0.31 | 0.10 | 0.08 | 0.09 | 10.6 | 10.6 | 10.5 |
| T-3 | 89.4 | 84.8 | 77.7 | 0.35 | 0.38 | 0.39 | 0.12 | 0.14 | 0.11 | 10.9 | 10.8 | 10.8 |
| T-2 | 86.1 | 85.7 | 81.2 | 0.38 | 0.35 | 0.44 | 0.15 | 0.12 | 0.14 | 11.4 | 10.7 | 10.9 |
| T-1 | 86.0 | 90.4 | 83.4 | 0.56 | 0.50 | 0.64 | 0.22 | 0.18 | 0.20 | 11.4 | 11.1 | 10.6 |
| T0 | 94.2 | 92.8 | 90.1 | 0.39 | 0.68 | 0.56 | 0.12 | 0.18 | 0.19 | 9.6 | 9.4 | 9.7 |
| T+1 | 93.7 | 93.3 | 80.9 | 0.43 | 0.57 | 0.57 | 0.14 | 0.19 | 0.17 | 10.8 | 10.5 | 10.6 |
| T+2 | 91.1 | 91.2 | 78.3 | 0.36 | 0.36 | 0.45 | 0.13 | 0.13 | 0.15 | 10.6 | 11.1 | 10.5 |
| T+3 | 89.5 | 93.1 | 85.4 | 0.27 | 0.30 | 0.37 | 0.11 | 0.10 | 0.12 | 10.8 | 10.3 | 10.5 |
| T+4 | 90.5 | 88.3 | 86.3 | 0.35 | 0.38 | 0.40 | 0.12 | 0.14 | 0.15 | 9.9 | 10.3 | 10.4 |
| T+5 | 89.8 | 88.6 | 89.4 | 0.43 | 0.53 | 0.31 | 0.14 | 0.17 | 0.10 | 9.5 | 9.5 | 9.9 |
| T+6 | 92.6 | 92.2 | 90.1 | 0.28 | 0.21 | 0.28 | 0.10 | 0.11 | 0.13 | 9.8 | 10.2 | 10.2 |
| SEM | 4.10 | 3.80 | 2.62 | 0.07 | 0.07 | 0.05 | 0.03 | 0.02 | 0.02 | 0.32 | 0.30 | 0.22 |
|  | Creatin kinase (U/L) | | | Creatinine (mg/dL) | | | Cu (μg/dL) | | | GGT (U/L) | | |
|  | NEFA-I | NEFA-II | NEFA-III | NEFA-I | NEFA-II | NEFA-III | NEFA-I | NEFA-II | NEFA-III | NEFA-I | NEFA-II | NEFA-III |
| T-7 | 181.2 | 184.8 | 180.8 | 1.66 | 1.78 | 2.00 | 234.7 | 315.3 | 352.2 | 27.2 | 30.3 | 24.8 |
| T-6 | 181.9 | 151.0 | 154.3 | 1.89 | 1.83 | 2.00 | 151.7 | 247.3 | 319.2 | 24.2 | 26.0 | 24.7 |
| T-5 | 172.9 | 139.6 | 195.3 | 2.01 | 1.95 | 1.92 | 150.8 | 268.6 | 257.9 | 22.0 | 23.6 | 23.7 |
| T-4 | 195.5 | 163.0 | 178.4 | 2.00 | 1.92 | 2.02 | 145.7 | 229.8 | 239.7 | 21.7 | 23.1 | 21.9 |
| T-3 | 183.4 | 215.9 | 215.8 | 1.99 | 1.95 | 2.12 | 159.3 | 232.9 | 262.4 | 21.3 | 21.3 | 20.4 |
| T-2 | 182.2 | 163.1 | 181.6 | 1.90 | 1.94 | 2.14 | 160.2 | 237.6 | 249.1 | 18.9 | 21.7 | 19.7 |
| T-1 | 184.8 | 142.2 | 164.2 | 1.85 | 1.86 | 2.10 | 150.1 | 231.2 | 199.9 | 21.6 | 21.4 | 19.6 |
| T0 | 155.3 | 175.0 | 195.0 | 2.05 | 2.09 | 2.11 | 55.0 | 86.6 | 90.7 | 18.1 | 20.2 | 17.5 |
| T+1 | 240.1 | 253.8 | 313.5 | 1.48 | 1.59 | 1.64 | 101.2 | 203.6 | 199.6 | 21.1 | 23.5 | 18.4 |
| T+2 | 186.2 | 204.7 | 225.3 | 1.40 | 1.47 | 1.58 | 152.4 | 242.7 | 224.7 | 21.9 | 23.8 | 19.2 |
| T+3 | 286.8 | 179.4 | 201.0 | 1.34 | 1.39 | 1.52 | 192.5 | 245.5 | 246.4 | 24.3 | 25.0 | 21.4 |
| T+4 | 252.7 | 199.9 | 253.5 | 1.29 | 1.37 | 1.51 | 207.0 | 286.8 | 258.8 | 27.4 | 26.4 | 23.5 |
| T+5 | 217.0 | 206.6 | 245.5 | 1.27 | 1.42 | 1.52 | 206.5 | 211.7 | 227.3 | 31.1 | 31.7 | 28.2 |
| T+6 | 225.9 | 208.1 | 203.3 | 1.28 | 1.25 | 1.38 | 160.9 | 246.5 | 206.0 | 29.7 | 33.1 | 26.9 |
| SEM | 40.9 | 38.4 | 27.8 | 0.08 | 0.08 | 0.06 | 37.5 | 35.3 | 25.6 | 1.84 | 1.73 | 1.26 |
|  | α-Globulin (g/dL) | | | LDH (U/L) | | | P (mg/dL) | | | Triglycerides (mg/dL) | | |
|  | NEFA-I | NEFA-II | NEFA-III | NEFA-I | NEFA-II | NEFA-III | NEFA-I | NEFA-II | NEFA-III | NEFA-I | NEFA-II | NEFA-III |
| T-7 | 1.49 | 1.58 | 1.55 | 1699.7 | 1689.0 | 1752.6 | 6.48 | 5.90 | 6.04 | 32.1 | 29.5 | 32.6 |
| T-6 | 1.53 | 1.50 | 1.55 | 1719.0 | 1602.6 | 1686.8 | 6.51 | 6.23 | 5.88 | 31.9 | 31.2 | 34.4 |
| T-5 | 1.31 | 1.36 | 1.47 | 1585.6 | 1373.2 | 1659.1 | 6.29 | 6.05 | 6.62 | 27.3 | 25.4 | 31.1 |
| T-4 | 1.38 | 1.35 | 1.37 | 1426.5 | 1345.3 | 1475.9 | 6.88 | 6.13 | 5.82 | 27.5 | 23.9 | 29.0 |
| T-3 | 1.38 | 1.44 | 1.39 | 1558.2 | 1427.0 | 1546.3 | 6.96 | 6.26 | 6.03 | 25.9 | 24.5 | 29.7 |
| T-2 | 1.31 | 1.34 | 1.39 | 1449.9 | 1378.7 | 1455.1 | 6.29 | 5.98 | 5.87 | 21.7 | 22.5 | 26.7 |
| T-1 | 1.27 | 1.28 | 1.31 | 1342.7 | 1260.8 | 1346.3 | 6.50 | 5.79 | 5.54 | 21.0 | 17.3 | 22.0 |
| T0 | 1.46 | 1.57 | 1.49 | 1478.2 | 1485.6 | 1445.1 | 5.37 | 4.86 | 4.99 | 23.7 | 22.7 | 24.3 |
| T+1 | 1.59 | 1.67 | 1.61 | 1370.4 | 1370.8 | 1491.8 | 5.99 | 5.71 | 5.37 | 16.3 | 18.1 | 16.8 |
| T+2 | 1.57 | 1.59 | 1.54 | 1531.6 | 1582.8 | 1560.3 | 5.94 | 5.54 | 5.41 | 18.9 | 21.3 | 18.8 |
| T+3 | 1.56 | 1.62 | 1.60 | 1661.7 | 1625.7 | 1544.9 | 6.04 | 5.24 | 5.48 | 18.7 | 18.3 | 20.2 |
| T+4 | 1.60 | 1.61 | 1.58 | 1575.1 | 1488.6 | 1566.2 | 5.42 | 5.43 | 5.37 | 20.4 | 20.0 | 20.1 |
| T+5 | 1.64 | 1.72 | 1.64 | 1560.8 | 1454.4 | 1445.1 | 5.58 | 5.31 | 5.48 | 20.5 | 21.5 | 24.2 |
| T+6 | 1.56 | 1.61 | 1.53 | 1736.3 | 1532.1 | 1593.6 | 5.80 | 5.28 | 5.29 | 20.3 | 20.5 | 18.7 |
| SEM | 0.05 | 0.05 | 0.03 | 74.2 | 69.8 | 50.5 | 0.24 | 0.23 | 0.16 | 1.92 | 1.80 | 1.31 |
|  | Urea (mg/dL) | | |  |  |  |  |  |  |  |  |  |
|  | NEFA-I | NEFA-II | NEFA-III |  |  |  |  |  |  |  |  |  |
| T-7 | 47.9 | 37.8 | 41.9 |  |  |  |  |  |  |  |  |  |
| T-6 | 33.9 | 30.9 | 35.1 |  |  |  |  |  |  |  |  |  |
| T-5 | 32.8 | 33.3 | 33.8 |  |  |  |  |  |  |  |  |  |
| T-4 | 30.8 | 32.1 | 30.4 |  |  |  |  |  |  |  |  |  |
| T-3 | 31.5 | 31.7 | 29.8 |  |  |  |  |  |  |  |  |  |
| T-2 | 27.9 | 26.1 | 28.9 |  |  |  |  |  |  |  |  |  |
| T-1 | 23.4 | 22.9 | 24.1 |  |  |  |  |  |  |  |  |  |
| T0 | 28.2 | 33.0 | 31.9 |  |  |  |  |  |  |  |  |  |
| T+1 | 50.2 | 44.1 | 44.6 |  |  |  |  |  |  |  |  |  |
| T+2 | 46.3 | 44.6 | 44.4 |  |  |  |  |  |  |  |  |  |
| T+3 | 47.3 | 47.0 | 45.6 |  |  |  |  |  |  |  |  |  |
| T+4 | 49.4 | 47.9 | 46.5 |  |  |  |  |  |  |  |  |  |
| T+5 | 52.5 | 47.8 | 51.1 |  |  |  |  |  |  |  |  |  |
| T+6 | 53.0 | 52.5 | 51.2 |  |  |  |  |  |  |  |  |  |
| SEM | 2.24 | 2.11 | 1.53 |  |  |  |  |  |  |  |  |  |
| NEFA-I, n=18: NEFA<0.29 mEq/L; NEFA-II, n=20: 0.29mEq/L≤NEFA<0.57mEq/L; NEFA-III, n=38: NEFA≥0.57mEq/L;  T-7: 7 weeks before calving; T-6: 6 weeks before calving; T-5: 5 weeks before calving; T-4: 4 weeks before calving; T-3: 3 weeks before calving; T-2: 2 weeks before calving; T-1: 1 week before calving; T0: calving; T+1: 1 week after calving; T+2: 2 weeks after calving; T+3: 3 weeks after calving; T+4: 4 weeks after calving; T+5: 5 weeks after calving; T+6: 6 weeks after calving; | | | | | | | | | | | | |
